# A Calcifying Odontogenic Cyst Associated with Compound Odontoma Mimicking a Tooth Germ

**DOI:** 10.1155/2021/9991772

**Published:** 2021-06-28

**Authors:** Eduardo Morato Oliveira, Lucas Alves da Mota Santana, Erika Rezende Silva, Leandro Napier Souza

**Affiliations:** ^1^Department of Oral Surgery and Pathology, School of Dentistry, Universidade Federal de Minas Gerais, Belo Horizonte, MG, Brazil; ^2^Department of Dentistry, Universidade Federal de Sergipe, Aracaju, SE, Brazil

## Abstract

Calcifying odontogenic cyst (COC) is a rare cyst that affects mainly the anterior region of the jaws. Generally, it appears as a unilocular radiolucent lesion containing peripheral foci of calcification, but with radiographic variations depending on the type of presentation. Here, we report an atypical case of COC associated with odontoma, initially diagnosed as a tooth germ, in the posterior region of the mandible of a 10-year-old male patient. Interestingly, the radiographic aspect appeared as a unilocular radiolucent lesion without peripheral foci of calcification in the edentulous region, having its size increased after traction of the impacted tooth adjacent to that area. Thus, the case presented in this study is aimed at calling dentists' attention to its developmental changes and related pathologies.

## 1. Introduction

Calcifying odontogenic cyst (COC) is a rare developmental cyst that favorably affects the anterior region of the jaws [1, 2]. It comprises less than 1% of intraosseous cysts arising in the maxillofacial complex, and it was recognized as a distinct pathological entity by Gorlin et al. in 1962 [3, 4]. Asymptomatic swelling, slow growth, and cortical expansion, accompanied or not by root resorption characterize the clinical picture of the cyst [5, 6].

Traditionally, COCs appeared as well-defined unilocular radiolucencies, exhibiting peripheral foci of calcification. Atypical radiographic presentations of COC are uncommon [7]. Here, we report a case of clinical and radiographic evolution of COC associated with odontoma, initially diagnosed as tooth germ in the posterior mandibular region. We also point out that dentists must draw their attention to its developmental changes and related pathologies to diagnose these cases accurately.

## 2. Case Report

**Figure 1:**
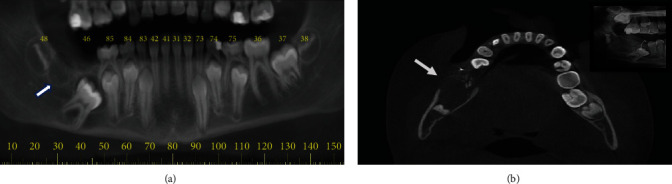
(a) Panoramic reconstruction of an initial CBCT showing a hypodense image (white arrow) in the region of a second molar suggesting the presence of a dental germ. (b) CBCT axial view showing the lesion expansion and cortical fenestration (white arrow). Foci of peripheral calcification in the lesion (in box).

A 10-year-old male patient was referred to orthodontic traction of an impacted right lower first molar (tooth 46). During clinical examination, no alteration was noted except the absence of tooth 46. No abnormalities were detected in previous medical history. A cone-beam computed tomography (CBCT) showed the presence of a radiolucent image measuring <1 cm located between teeth 46 and 48, which was initially interpreted as a dental germ (Figure 1(a)). The patient was submitted to surgery under local anesthesia to achieve tooth crown exposure and bonding of an orthodontic device to traction of its right lower first molar. Six months after the procedure, in a routine panoramic radiograph during orthodontic treatment, a well-defined radiolucent image with radiopaque foci was identified in the region of the suggestive dental germ. Additionally, an increasing volume with cortical expansion and fenestration was observed in a CBCT investigation (Figure 1(b)).

**Figure 2:**
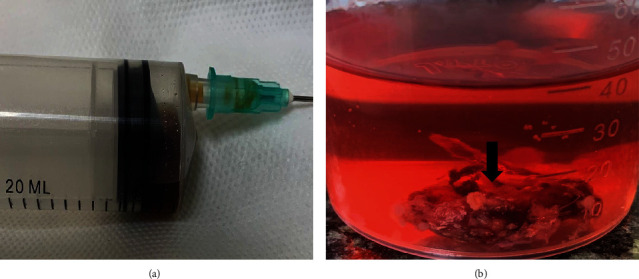
(a) Citrus yellow-colored liquid obtained after aspiration puncture of the lesion. Suggestive alteration of odontogenic nature. (b) Anatomopathological specimen obtained after biopsy. Presence of cystic cavity with fibrotic capsule in the lesion (black arrow).

**Figure 3:**
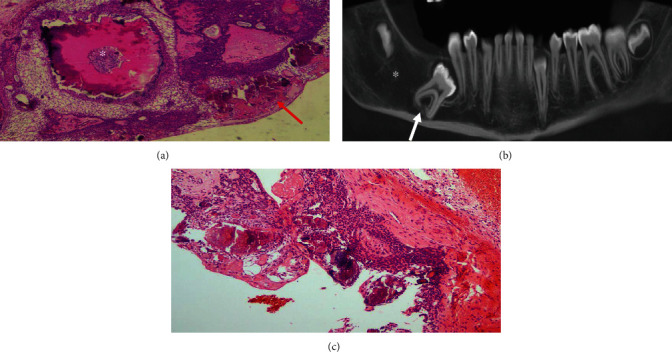
(a) Photomicrograph stained by hematoxylin-eosin (200x) revealing features of a calcifying odontogenic cyst with presence of ghost cells (red arrow) associated with odontoma (asterisk). (b) Photomicrography showing a cystic lesion with a fibrous tissue capsule and lining of odontogenic epithelium of ameloblastic type loosely arranged with several amounts of ghost cells (200X). (c) Panoramic reconstruction of a control CBCT evidencing a new bone formation in the area (asterisk). Impacted first molar (white arrow).

Thereby, a provisional diagnostic hypothesis of odontogenic lesions was made, suggesting adenomatoid odontogenic tumor and COC. The chosen clinical conduct was the surgical enucleation of the lesion. A straight incision was made in the alveolar ridge with flap detachment to facilitate access to the lesion, and an aspiration puncture was performed revealing a liquid content (Figure 2(a)). During surgery, the lesion was completely detached from the mandibular bone, allowing visualization of a capsule with a cystic aspect (Figure 2(b)). The specimen was sent for histopathologic examination, and the microscopic findings revealed a cystic cavity lined by a thin odontogenic epithelium with ameloblastomatous features. Ghost cells and calcification foci were also observed. Furthermore, the formation of dentinoid matrix, immature enamel, and fibrous connective tissue was noticed. Therefore, the diagnosis of COC associated with odontoma was established (Figures 3(a) and 3(b)). One year later, no signs of recurrence were observed and a CBCT revealed new bone formation in the region (Figure 3(c)). Currently, the patient is undergoing clinical and radiographic follow-up with pediatric dentistry specialists and orthodontists for adequate establishment of occlusal dynamics.

## 3. Discussion

Although COC is a benign lesion and a pathological entity widely known, its occurrence is considered uncommon [1]. Generally, patients are asymptomatic and major clinical signs include alveolar expansion, cortical perforation, and root resorption of adjacent teeth [2]. From all COCs arising in the mandible, about 65% is found in the anterior region in between canines. Approximately, 10-32% of cases are associated with unerupted teeth (especially canine) [1]. Although the age range of COC involvement is wide, Arruda et al.^2^ reinforce that one-third of patients are affected in childhood and adolescence. The case reported herein is in accordance with the literature.

Commonly, COC appears as a unilocular radiolucent area with well-defined limits and may exhibit small radiopaque deposits of various aspects [2, 7]. In some cases, the radiographic aspect of COC may be masked, primarily when developing as periapical radiolucent areas. Thus, the differential diagnosis can be established with other lesions of different radiographic aspects, such as ameloblastoma, odontogenic keratocysts, periapical cyst, ameloblastic fibroodontoma, and adenomatoid odontogenic tumor [2, 4, 6, 8].

In the current case, the lesion initially appeared as a well-delimited radiolucent area in the edentulous mandibular bone near an unerupted molar, which did not resemble a COC. During the attempt to traction the impacted tooth, an increasing volume with cortical expansion and fenestration were observed surrounding the radiolucent area, raising the possibility of an odontogenic lesion. Curiously, the lesion resembled a tooth germ in the crypt stage. Due to the exceptionality of the case, a vast literature search was carried out, and to our knowledge, this is the first report of its kind related to COC. On the other hand, the unexpected histological result only increases the notoriety of this report; once, authors described that some cases of delayed eruption may be attributed to physiological disorders impacting interruptions on Nolla's stages [9], thus leading to the most plausible initial diagnosis of a tooth germ, as initially presumed in this report.

Regarding that, the literature argues that the odontogenic tumors can share the same structures of a tooth germ, which is difficult to diagnose with immature structures of dental tissue, especially in children and adolescents [10]. For this reason, the knowledge of histological and radiological aspects of both developing teeth and odontogenic neoplasms is required to the establishment of an accurate clinical conduct.

This comparative analysis is crucial and may avoid diagnostic mistakes to several oral lesions (odontogenic or nonodontogenic origin), especially when malignant lesions are underdiagnosed. In a retrospective study conducted by Bacci et al., [11] the authors reported that upon evaluation of 1,566 samples of biopsy, an erroneous diagnosis between the radiographic examination and the histological analyses was found in 31.5% of the cases. Among such cases, over 80% of these records correspond to malignant or potentially malignant lesions. In another work, Almazrooa et al. [12] reported that the rate of concordance between histopathologic findings and radiographic interpretation of jaw lesions was lower than 50%. Consequently, such diagnostic misunderstandings may influence the treatment of patients as well as prognosis values in cases of malignancy. Therefore, a histologic examination must be performed in excised tissues and particularly those in which radiographic characteristics may generate confusion.

Also, it is important to highlight that COC may present wide-ranging radiographic patterns, as discussed by different studies. One study by Lida et al.^7^ investigated the radiographic profile of 11 cases of COC and only one of them arose from the edentulous mandible. Arruda et al.^2^ debated that radiolucent COCs may undergo calcification and progress to a lesion of mixed radiographic appearance, thus causing swelling and cystic expansion. Also, some works suggest that the remaining odontogenic epithelial cords when stimulated may induce the formation of dental tissue in the adjacent connective tissue wall [13].

The most remarkable feature of COC is the presence of ghost cells in an ameloblastoma-like epithelium [4, 14]. In approximately 22% of diagnosed cases, an association with odontoma is seen [15]. Although the origin of the COC is linked to the remnants of the dental lamina [14], recent data suggest that most COC carry a mutation in the CTNNB1 gene responsible for beta-catenin protein synthesis [16]. For most COCs (specifically those with smaller lengths), enucleation is the treatment of choice, with <5% of cases developing a recurrence [17].

As a result, a CBCT evolution of a COC in the mandibular molar area was described, going from a hypodense profile to a mixed appearance, thus calling the attention of dentists that radiographic or tomographic images may lead to misinterpretations between odontogenic lesions and dental apparatus forming structures.

## Data Availability

Access to data is restricted due to patient's privacy.

## References

[1] Kler S., Palaskar S., Shetty V. P., Bhushan A. (2009). Intraosseous calcifying cystic odontogenic tumor. *Journal of Oral and Maxillofacial Pathology*.

[2] de Arruda J. A. A., Schuch L. F., Abreu L. G. (2018). A multicentre study of 268 cases of calcifying odontogenic cysts and a literature review. *Oral Diseases*.

[3] Gorlin R. J., Pindborg J. J., Clausen F. P., Vickers R. A. (1962). The calcifying odontogenic cyst--a possible analogue of the cutaneous calcifying epithelioma of Malherbe: an analysis of fifteen cases. *Oral Surgery, Oral Medicine, and Oral Pathology*.

[4] Gadipelly S., Reddy V. B., Sudheer M., Kumar N. V., Harsha G. (2015). Bilateral calcifying odontogenic cyst: a rare entity. *Journal of Maxillofacial and Oral Surgery*.

[5] Irani S., Foroughi F. (2017). Histologic variants of calcifying odontogenic cyst: a study of 52 cases. *The Journal of Contemporary Dental Practice*.

[6] Rojo R., Prados-Frutos J. C., Gutierrez Lázaro I., Herguedas Alonso J. Á. (2017). Calcifying odontogenic cysts. *Journal of Stomatology, Oral and Maxillofacial Surgery*.

[7] Iida S., Fukuda Y., Ueda T., Aikawa T., Arizpe J. E., Okura M. (2006). Calcifying odontogenic cyst: radiologic findings in 11 cases. *Oral Surgery, Oral Medicine, Oral Pathology, Oral Radiology, and Endodontics*.

[8] de Carvalhosa A. A., de Araújo Estrela C. R., Borges A. H., Guedes O. A., Estrela C. (2014). 10-year follow-up of calcifying odontogenic cyst in the periapical region of vital maxillary central incisor. *Journal of Endodontia*.

[9] Klein O. D., Oberoi S., Huysseune A., Hovorakova M., Peterka M., Peterkova R. (2013). Developmental disorders of the dentition: an update. *American Journal of Medical Genetics. Part C, Seminars in Medical Genetics*.

[10] Slootweg P. J. (2007). Update on tooth formation mimicking odontogenic neoplasia. *Head and Neck Pathology*.

[11] Bacci C., Donolato L., Stellini E., Berengo M., Valente M. (2014). A comparison between histologic and clinical diagnoses of oral lesions. *Quintessence International*.

[12] Almazrooa S., Binmadi N. O., Khalifa H. M., Jadu F. M., Jan A. M., Meisha D. E. (2019). The agreement rate between radiographic interpretation and histopathologic diagnosis of jaw lesions. *Radiology research and practice*.

[13] Neelima A. M. (2012). *Textbook of Oral and Maxillofacial Surgery*.

[14] El-Naggar A. K., Chan J. K. C., Takata T., Grandis J. R., Slootweg P. J. (2017). The fourth edition of the head and neck World Health Organization blue book: editors' perspectives. *Human Pathology*.

[15] Ledesma-Montes C., Gorlin R. J., Shear M. (2008). International collaborative study on ghost cell odontogenic tumours: calcifying cystic odontogenic tumour, dentinogenic ghost cell tumour and ghost cell odontogenic carcinoma. *Journal of Oral Pathology & Medicine*.

[16] Yukimori A., Oikawa Y., Morita K. I. (2017). Genetic basis of calcifying cystic odontogenic tumors. *PLoS One*.

[17] Buchner A. (1991). The central (intraosseous) calcifying odontogenic cyst: an analysis of 215 cases. *Journal of Oral and Maxillofacial Surgery*.

